# Phenotype-Based HPLC-Q-TOF-MS/MS Coupled With Zebrafish Behavior Trajectory Analysis System for the Identification of the Antidepressant Components in Methanol Extract of Anshen Buxin Six Pills

**DOI:** 10.3389/fphar.2021.764388

**Published:** 2021-11-22

**Authors:** Jiani Liu, Yue Shang, Juanlan Xiao, Huirong Fan, Min Jiang, Saijun Fan, Gang Bai

**Affiliations:** ^1^ State Key Laboratory of Medicinal Chemical Biology, College of Pharmacy and Tianjin Key Laboratory of Molecular Drug Research, Nankai University, Tianjin, China; ^2^ Institute of Radiation Medicine, Chinese Academy of Medical Sciences and Peking Union Medical College, Tianjin, China; ^3^ Graduate School, Tianjin University of Traditional Chinese Medicine, Tianjin, China; ^4^ The Institute of Radiation Medicine, Chinese Academy of Medical Sciences, Tianjin, China

**Keywords:** Anshen Buxin Six Pills, anti-depression, costunolide, dehydrocostus lactone, phenotype-based screening method

## Abstract

Phenotype screening has become an important tool for the discovery of active components in traditional Chinese medicine. Anshen Buxin Six Pills (ASBX) are a traditional Mongolian medicine used for the treatment of neurosis in clinical settings. However, its antidepressant components have not been explicitly identified and studied. Here, the antidepressant effect of ASBX was evaluated in adult zebrafish. High performance liquid chromatography-mass spectrometry (HPLC-Q-TOF-MS/MS) was combined with zebrafish behavior trajectory analysis to screen and identify the antidepressant-active extract fraction and active components of ASBX. Finally, the antidepressant effect of the active ingredients were verified by the behavior, pathology, biochemical indices and protein level of adult fish. The novel tank driving test (NTDT) showed that ASBX can effectively improve the depressive effect of reserpine on zebrafish. Petroleum ether and dichloromethane extracts of ASBX were screened as antidepressant active extracts. Costunolide (COS) and dehydrocostus lactone (DHE) were screened as the active components of ASBX. COS had been shown to significantly improve the depressive behavior, nerve injury and neurotransmitter levels (5-hydroxytryptamine (5-HT) and norepinephrine (NE)) of zebrafish by inhibiting the high expression of serotonin transporter and norepinephrine transporter induced by reserpine suggesting the antidepressant effect of COS may be related to its effect on 5-HT and NE pathways. This study provided a phenotype based screening method for antidepressant components of traditional Chinese medicines, so as to realize the separation, identification and activity screening of components at the same time.

## Introduction

Traditional Chinese medicines (TCM) have been shown to have an important role in the treatment of central nervous system (CNS) diseases (Li et al., 2016). As a result, the active components of TCM have become important sources of lead compounds (Gao et al., 2019). High-performance liquid chromatography-mass spectrometry (HPLC-Q-TOF-MS/MS) technology combined with target-based (Hou et al., 2020) and cell-based activity detection methods (Zhang et al., 2021), has been widely used in screening for lead compounds. However, it is not suitable for screening active ingredients where the target is unknown. Due to the complexity of the mechanism of central nervous system diseses (Krahn et al., 2020), such as depression, the screening methods based on cells and targets can not show the complex physiological activities of the nervous system. Therefore, phenotypic screening is more advantageous because it can detect the activity of drugs at the complete animal level (Henry and Wlodkowic, 2019). However, the traditional mammalian based screening method has the defects of large dosage and can not screen a large number of samples quickly. Therefore, a rapid screening method of neuroactive drugs needs to be established.

Depression has become one of the most common mental diseases (Yao et al., 2019). Monoamine metabolites levels, ability to cross the blood–brain barrier, and neuroprotective activity have been used as screening indices for antidepressant components (Zhang et al., 2019). Virtual screening methods, based on correlation analysis of metabolites and network pharmacology (Liu et al., 2021) with molecular docking technology (Dhiman et al., 2018) can predict the active ingredients of antidepressants. However, these methods cannot directly evaluate the efficacy of ingredients, and therefore, they may give false-positive and false-negative results. In recent years, more and more attention has been paid to phenotype-based drug screening, which has become an important method of early drug discovery. These methods, however, cannot directly evaluate the efficacy of ingredients, and therefore, there may be false-positive and false-negative results. In recent years, more and more attention has been paid to phenotype-based drug screening, which has become an important method of early drug discovery (Kuusanmaki et al., 2020). Neuroactive molecules usually have the characteristics of pleiotropy, phenotype-based screening methods can more directly reflect the pharmacological effects of chemical components in the whole organism, without assuming relevant molecular targets (Henry and Wlodkowic, 2019). Therefore, it is necessary to establish a high-throughput, phenotype-based screening method to identify new neuroactive compounds present in TCMs. Zebrafish larvae are important tool for screening large-scale components and discovering new neuroactive drug, as a phenotype-based drug screening model, because their advantages of requiring a low dosage of drug, exhibiting fast reproduction (Stewart et al., 2015) and homology with human genes in the monoaminergic nervous system (Aslanzadeh et al., 2019). The zebrafish swimming behavior-based screening method has been widely used in the exploration of mental diseases, such as Alzheimer’s disease (Li et al., 2020).

Anshen Buxin Six Pills is a traditional Mongolian medicine preparation that has been, and still is clinically used to treat neurosis. Therefore, it has potential as a library of lead compounds used in the treatment of depression and other psychiatric diseases. It is composed of six traditional medicines, including the root of *A* *ucklandia costus* Falc., the seed of *Myristica fragrans* Houtt., the fruit of *Choerospondias axillaris* (Roxb.) B.L.Burtt & A.W.Hill., the flower of *Syzygium aromaticum* (L.) Merr. & L.M.Perry., the resin of *Liquidambar formosana* Hance. and the heart of Asian water buffaloes (*Bubalus bubalis* Linnaeus) or yellow cattle (*Bos taurus domesticus* Gmelin). The content radio of these six traditional Chinese medicines is the same, and the content is 100 g individually, the preparation method is crushed into fine powder, sieved, mixed evenly, pan pills with water, coated with 0.2 g cinnabar, polished and dried. Costunolide (COS) and dehydrocostus lactone (DHE) have been identified as the main active components of *Aucklandia costus* Falc (Dong et al., 2018). However, the antidepressant active components of the Anshen Buxin Six Pills are not clear.

In this study, the strategy of HPLC-Q-TOF-MS/MS technology combined with a zebrafish behavior trajectory analysis system is established to screen for antidepressant components of ASBX. This method can theoretically establish a phenotype-based screening platform to achieve the separation, identification, and activity screening of TCMs at the same time.

## Materials and Methods

### Preparation of Plant Extracts

Anshen Buxin Six Pills (Lot number: 180908) was purchased from Ulanhot Sino-Mongolian Pharmaceutical Co. Ltd. (Ulanhot, Inner Mongolia, China). Anshen Buxin Six Pills powder (10 g) was dissolved in 100 ml of methanol and extracted via ultrasonication of 600 W for 1 h using a ultrasonic cleaning machine (SB-25-12-DT), which was purchased from Ningbo Scientz Biotechnology Co.,Ltd. (Ningbo, Zhejiang, China). After filtration, the methanol extract of Anshen Buxin Six Pills (ASBX) was obtained via rotary evaporation. The solvent of the extract was removed by the oil pump and vacuum freeze-dried into powder to obtain the lyophilized powder of ASBX, which was stored in −20°C for the next experiment. Follow the above steps to get ASBX, then, the ASBX were heated with water in 100 ml and resuspended. Then gradient extraction was carried out, each solvent was 300 ml each time, extracted for 3 times, and the solvent was removed by rotary distillation, petroleum ether extract (PEE), dichloromethane extract (DME), ethyl acetate extract (EAE), water-saturated *n-*butanol extract (BUE), and aqueous extracts (AQE) were obtained. Finally, the solvent was evaporated by oil pump and vacuum freeze-dried to obtain the samples of each extraction layer.

### Chemicals and Reagents

Reserpine (83580, purity ≥ 99.0%) was purchased from Sigma-Aldrich (St. Louis, MO, United States). Fluoxetine (FLX, F830634, purity ≥98.0%) was purchased from Shanghai Macklin Biomedical Co., Ltd. (Shanghai, China), and Costunolide (COS, AB0612, purity ≥98.0%) was purchased from Chengdu Alfa Biotechnology Co., Ltd. (Chengdu, Sichuan, China). Dehydrocostus lactone (DHE, D91141, purity ≥98.0%) was purchased from Shanghai Acmec Biochemical Co. Ltd. (Shanghai, China). ELISA kit for 5-hydroxytryptamine (5-HT) and dopamine (DA) was purchased from Wuhan Genomei Technology Co., Ltd. (Wuhan, Hubei, China). ELISA kit for norepinephrine (NE), serotonin transporter (SERT) and norepinephrine transporter (NET) was purchased from Shanghai Jianglai Industrial Co., Ltd. (Shanghai, China).

### Experimental Animals

Adult zebrafish (AB strains, 5 months-old, male:female, 1:1) were purchased from Shanghai FishBio Co. Ltd. (Shanghai, China) and acclimated in fish tanks purchased from Shanghai Haisheng Biotech Co., Ltd. (Shanghai, China) with a recirculating aquatic system. The system water contained KCl 0.05 g/L, NaHCO_3_ 0.025 g/L, NaCl 3.5 g/L and CaCl_2_ 0.1 g/L. The zebrafish embryos were incubated with Holt buffer (15 mM NaCl, 0.67 mM KCl, 0.03 mM NaHCO_3_, 0.90 mM CaCl_2_, pH 7.2). Adult zebrafish and zebrafish embryos were maintained under a photoperiod of 14 h light/10 h dark. All the animal studies have been approved by the Tianjin University of Traditional Chinese Medicine of Laboratory Animals Care and Use Committee (TCM-LAEC2016032).

### Establishment of Depression Adult Zebrafish Model and Experimental Grouping of ASBX

The configuration of solutions of ASBX groups was the lyophilized powder of ASBX was dissolved with DMSO, and dilute to 0.8% DMSO with system water. The experimental grouping is described as follows. To investigate the antidepressant activity of ASBX, adult zebrafish were randomly divided into six groups (n = 10): control group (Con, 0.8% DMSO system water), model group (Mod, 0.8% DMSO system water), Fluoxetine group (FLX, 10 μmol/kg), and ASBX groups (ASBX-80 mg/kg, ASBX-20 mg/kg, and ASBX-5 mg/kg). The FLX group was used as the positive control. Except for the Con group, zebrafish were soaked with reserpine (40 μg/ml) for 1 h every day for 7 days to induce a depressive phenotype (Tang et al., 2019). After treatment with reserpine daily, adult zebrafish were injected intraperitoneally with the above sample solutions.

### Novel Tank Diving Test

The experimental procedure for the NTDT is described below. The experiment was conducted according to the protocol (Liu et al., 2020), with little change. The part of the tank with water was equally divided into two analysis areas, which was defined as top and bottom. After the zebrafish adapted to the tank environment for 5 min, the swimming trajectory of zebrafish in the first 10 min was recorded and analyzed using Noldus Ethvision XT 15 software (Noldus Information Technology, Leesburg, VA, United States). The following parameters were measured: total distance traveled (cm), average velocity (cm/s), freezing duration(s), number of transitions from bottom to top, first latency to enter the top area (s), and time spend in the top area.(s).

### Nissl Staining and Determination of Content of Neurotransmitter

The experimental procedure for Nissl staining and the determination of neurotransmitter content are described below. The brains of the zebrafish were stained with Nissl stain solution (Beijing SolarBio Science & Technology Co., Ltd., Beijing, China) according to the manufacturer’s instructions, and digital images were taken with a Nikon CiS microscope (Nikon). Image-Pro Plus software (version 6.0) was used to analyze the images and count the number of Nissl-positive cells in the periglomerular gray zone (PGZ) and ventrolateral nucleus of the semicircular torus (TSvl). The concentrations of 5-HT, DA, and NE in the brain tissues of zebrafish were detected using Enzyme-linked immunosorbent assay (ELISA) kits for 5-HT, DA and NE, according to the manufacturer’s instructions.

### Establishment of Depression Zebrafish Larvae Model and Preparation of Different Extraction Layers and Components of ASBX

The light/dark preference test was used to examine the effects of the extract layer and components of ASBX on reserpine-induced depression-like behavior in zebrafish larvae, analyzed by the zebrafish behavior trajectory analysis system. Reserpine was dissolved in 3 ml of Holt buffer containing 0.1% DMSO. The embryos (30/plate) were kept in 12-well plates with Holt buffer containing reserpine (4 μg/ml) or 0.1% DMSO Holt Buffer (Con group) at 6 h post fertilization and maintained in the dark for 6 days to obtain zebrafish larvae for the test. The establishment of depression phenotype of zebrafish larvae was referred to the methods (Wang et al., 2019) in the literature.

Freeze-dried extraction layers different extraction layers (PEE, DME, EAE, BUE, AQE) and lyophilized powder of ASBX were dissolved with DMSO and diluted with Holt buffer to the content of DMSO was 0.1%. The experimental groups are described as follows. To screen the anti-depression extraction layer(s) in ASBX, zebrafish larvae treated with reserpine were transferred to solutions of different extraction layers (PEE, DME, EAE, BUE, AQE, 80 μg/ml), ASBX methanol extract (80 μg/ml), Mod (0.1% DMSO Holt Buffer), and FLX (Fluoxetine, 10 μM) in 12-well plates. The actual drug amount of each extraction layer is calculated according to the equivalent crude drug amount, indicating the proportion of substances in each extraction layer in ASBX of 80 μg/ml. The FLX group was used as the positive control. The fractions (1–46 min) of PEE (10 mg/ml) from HPLC were collected into 10 ml EP tubes every 2 min for 23 fractions total. The contents of the EP tubes were then evaporated to dryness in a vacuum drying oven and dissolved in 3 ml Holt buffer. A sample that matches a peak of the corresponding time in the chromatogram is considered one component. COS and DHE groups (0.1, 1 and 10 μmol/L) were used to verify the antidepressant activity of the screened components from the light/dark preference test.

### Light/Dark Preference Test on Zebrafish Larvae

The light/dark preference test was then conducted according to the protocol (Wang et al., 2019) with some modifications. Briefly, the culture medium with reserpine of zebrafish larvae was replaced with drug solutions, soaked for 2 h. Zebrafish larvae treated with above mentioned drug solutions were transferred to each well of a 96-well plate (length × width: 125 × 85 mm, well diameter 10 mm), with 300 μl of Holt buffer, and adapted for 3 min before the test.

The light conditions are described as follows. The setting of the light and dark stimulus program was started 5 min after beginning video recording of the behavior trajectory. For light stimulation, the program was set as follows: light was applied for 3 min, off for 3 min, and repeated for three cycles. The video recordings were analyzed using Noldus Ethovision XT 15 software (Noldus Information Technology, Leesburg, VA, United States) by tracking the center of mass of the individual fish over time. Parameters were measured as the total distance traveled (mm) and average velocity (mm/s).

### HPLC-Q-TOF-MS/MS Analysis of PEE

After determining PEE was the active extraction layer, PEE was dissolved in methanol with concentration of 0.1 mg/ml, then subjected to HPLC-Q-TOF-MS/MS analysis. A diamonsil^®^ C18(2) (250 × 4.6 mm, 5 μm) column was used to separate and detect the components of ASBX extraction, with the column temperature maintained at 40°C. The mobile phase was a gradient elution system of A (CH_3_OH) and B (HCOOH:H_2_O = 0.05:100), and elution was performed at a flow rate of 0.5 ml/min. The injection volume was 10 μl, and the gradient duration program was 0–2 min, 96% B; 2–6 min, 96–35% B; 6–41 min, 35%–0% B; 41–48 min, 0% B; 48–48.5 min, 0%–96% B; 48.5–56 min, 96% B.

Accurate mass measurements were collected using an AB Sciex Triple-TOF™ 4600^+^ mass spectrometer (Framingham, MA, United States) with an electrospray ionization (ESI) system. The ESI/MS spectra were acquired in the positive ion mode, and the ionizing voltage was set to 5,500 V. The temperature of the source was 300°C. The sample cone voltage was set to 30 V. High-purity nitrogen was used for nebulization and as the auxiliary gas. Ion source gases (N_2_) 1 and 2 were maintained at 60 psi, and the curtain gas (N_2_) pressure was maintained at 35 psi. The relative impact energy was 10 eV. The de-clustering voltage was 40 V. Peak View 2.2 was employed to process the data.

### Experimental Grouping of COS in NTDT

To study the antidepressant activity of COS, adult, reserpine-treated zebrafish were randomly divided into six groups (n = 10): control group (Con, 0.8% DMSO system water), model group (Mod, 0.8% DMSO system water), FLX group (Fluoxetine, 10 μmol/kg), and COS groups (COS-10 μmol/kg, COS-40 μmol/kg, and COS-160 μmol/kg). Other experimental conditions were the same as those described in section *Novel Tank Diving Test*.

### Protective Effect of COS on Reserpine-Induced Neuronal Injury

The experimental conditions for Nissl staining and determination of the content of neurotransmitters (5-HT, DA, and NE) were the same as in Section *Nissl Staining and Determination of Content of Neurotransmitter*.

### Determination of Expression Level of Monoamine Transporter

The expression levels of SERT and NET were quantified using ELISA kits (Shanghai Jianglai Industrial Co., Ltd., Shanghai, China) according to the manufacturer’s instructions.

### Statistical Analysis

GraphPad Prism Software (version 6.0) was used for the statistical analysis of results, and one-way analysis of variance was applied for data analysis, followed by the Tukey’s test for comparison of significant differences between the means. The results are expressed as the mean ± SEM. Statistical significance was set at *p* < 0.05.

## Results

### Anti-Depressive Effect of ASBX on Zebrafish Behavior

**FIGURE 1:**
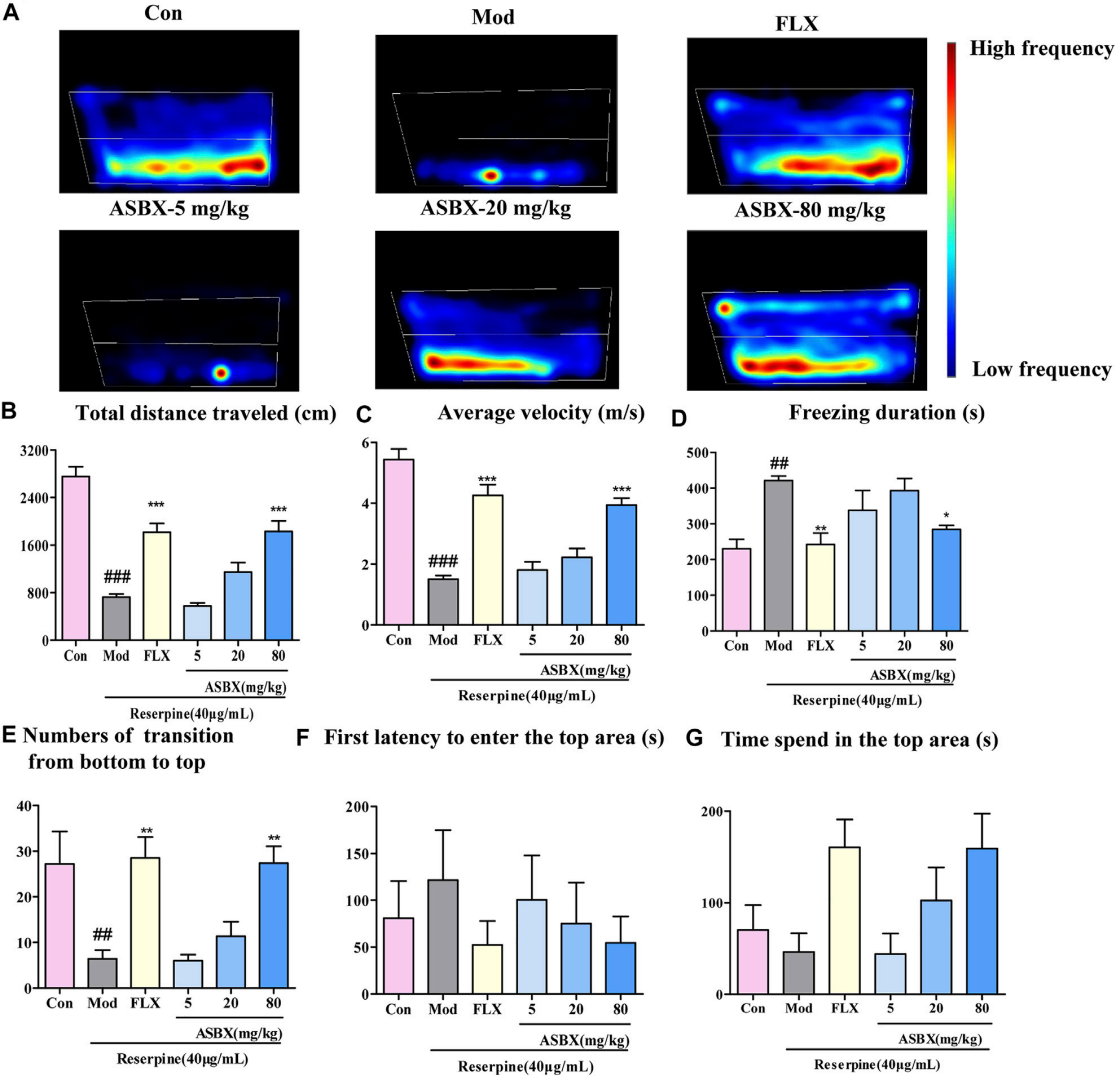
Antidepressant effect of Anshen Buxin Six Pills (ASBX) was tested using a novel tank diving test. **(A)** Heat maps of the behavior trajectory. The bar chart shows the swimming ability, exploratory behavior, and freezing behavior of adult zebrafish. **(B)** Total distance travelled (cm) **(C)** Average velocity (cm/s) **(D)** Freezing duration (s) **(E)** Number of transitions from bottom to top **(F)** First latency to enter top area (s) **(G)**. Time spend in the top area. Data are expressed as the mean ± SEM) (n = 10/group). Significance was defined as ^##^ *p* < 0.01, ^###^ *p* < 0.001 compared with Con group, ^*^ *p* < 0.05, ^**^ *p* < 0.01, ^***^ *p* < 0.001 compared with Mod group.

To study the antidepressant effect of ASBX on zebrafish behavior, a NTDT was conducted. The NTDT takes advantage of the stress response of zebrafish in a new environment, which can be used to simulate anxiety, depression, and other behaviors (Benneh et al., 2017). As shown in the heat map in Figure 1A, exposure to reserpine resulted in low-frequency swimming at the top of the tank (simulating the effects of depression); this behavior was reversed by ASBX and FLX. Compared with the model group, the ASBX-80 mg/kg and FLX groups showed significantly improved swimming ability (total distance traveled and average velocity) (^***^ *p* < 0.001, Figures 1B,C), freezing behavior (shortened freezing time) (^*^ *p* < 0.05, Figure 1D), and exploration behavior (increased transitions from bottom to top and shortened first latency to top area) (^*^ *p* < 0.05, Figure 1E). But there was no siginificant diffrences in First latency to enter the top area and Time sped inthe top area (Figures 1 F,G).

### Protective Effect of ASBX on Reserpine-Induced Neuronal Injury

**FIGURE 2:**
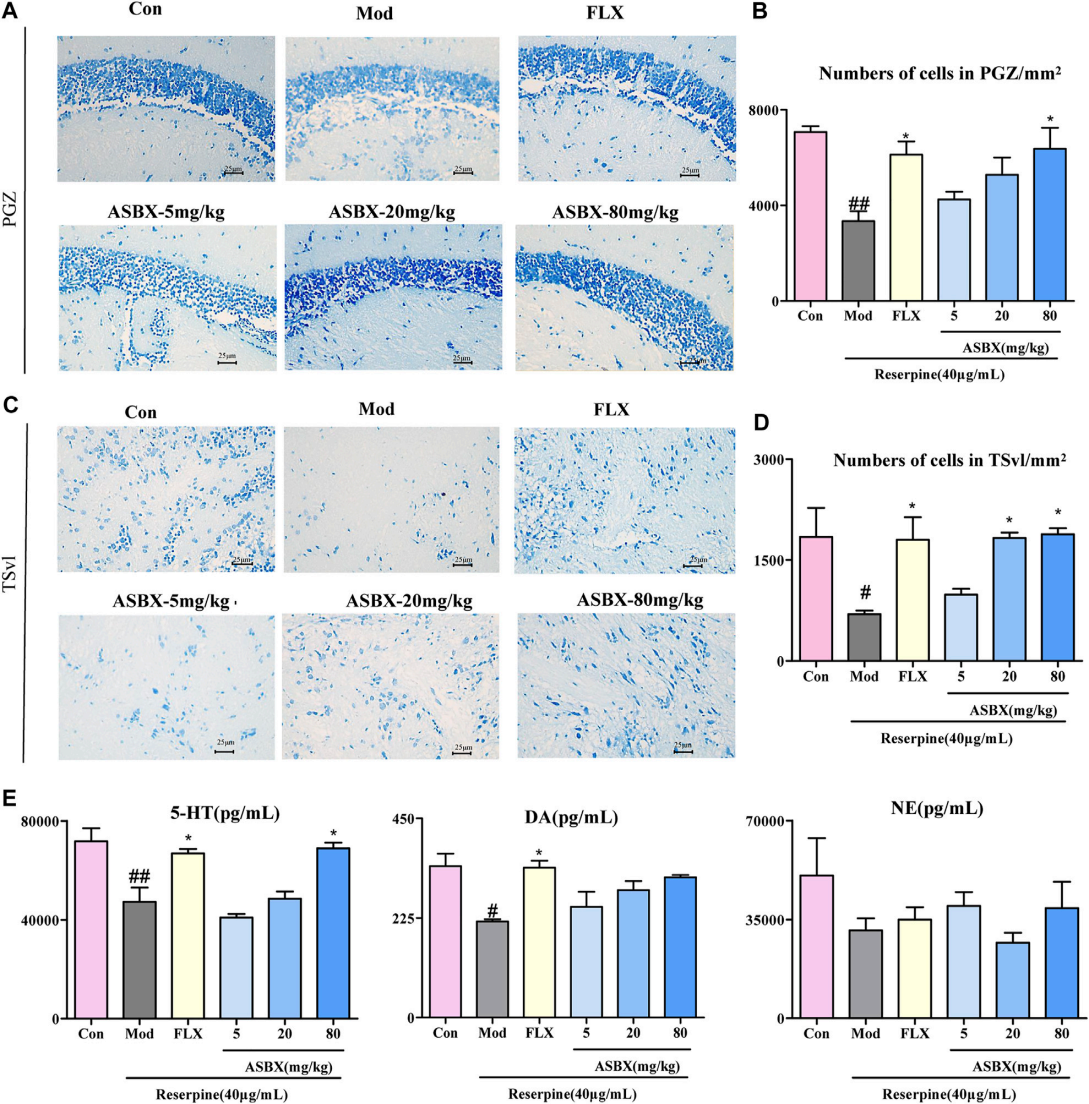
Protective effect of Anshen Buxin Six Pills (ASBX) on reserpine-induced neuronal injury. **(A)**. Nissl stain of zebrafish brain (400×) in periglomerular gray zone (PGZ) **(C)**. Nissl stain of zebrafish brain in ventrolateral nucleus of the semicircular torus (TSvl). **(B and D)** are the quantitative statistics of neurons in the PGZ and TSvl, respectively. **(E)**. The content of 5-HT, DA, NE in zebrafish brain was measured by ELISA. Data are expressed as the mean ± SEM (n = 3/group). Significance was defined as ^#^ *p* < 0.05, ^##^ *p* < 0.01, ^*^ *p* < 0.05.

Based on the improved effect of ASBX on the swimming behavior of zebrafish, we further verified its antidepressant effect at the level of histopathology and biochemical indexes by Nissl staining and enzyme-linked immunosorbent assay (ELISA). Neuronal regeneration dysfunction is one cause of depression (Fang et al., 2018). Exposure to reserpine led to the reduction and disordered arrangement of Nissl bodies in cytoplasm, the distance between cells became larger, and the boundary is not clear in periglomerular gray zone (PGZ) (Figures 2A,C), conversely, ASBX-80 mg/kg and FLX groups reversed the reduction in the number of neurons in the PGZ and TSvl induced by reserpine, there are significant differences in statistical results.(^*^ *p* < 0.05, Figures 2B,D).

Neurotransmitters are closely associated with the neurobiological mechanisms of depression (Liu et al., 2018). ASBX-80 mg/kg and FLX groups significantly reversed the reduction in the levels of 5-HT (^*^ *p* < 0.05) after exposure to reserpine (Figure 2E).

### Discovery of Antidepressant Components of ASBX Through HPLC-Q-TOF-MS/MS

**FIGURE 3:**
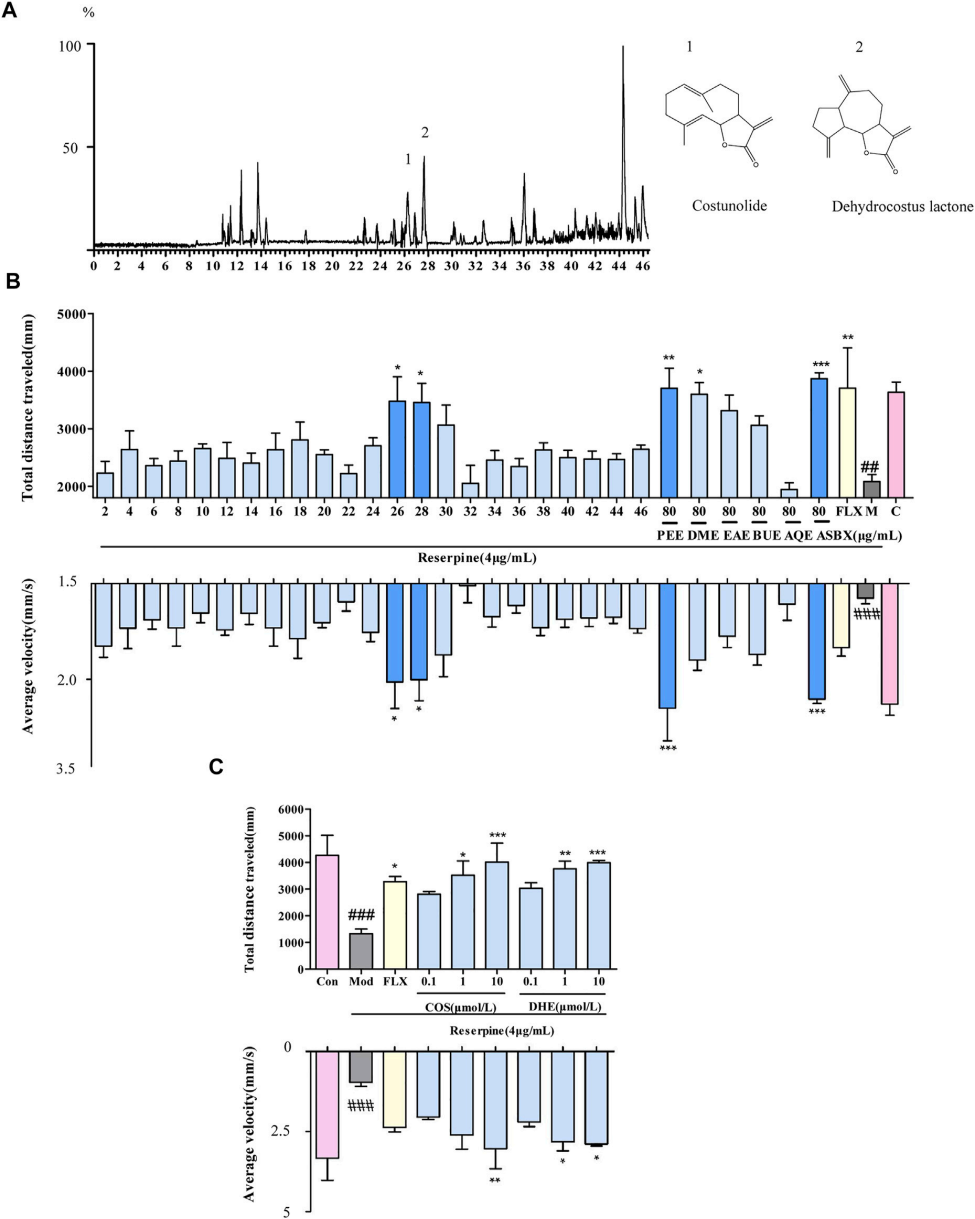
Screening for the antidepressant components of methanolic extract of Anshen Buxin Six Pills (ASBX) on the swimming ability of zebrafish larvae. **(A)**. Total ion chromatograms (TIC) of the petroleum ether extract (PEE) of ASBX in positive ESI mode **(B)**. Bioactivity histogram obtained via zebrafish behavior trajectory analysis system are analyzed as total distance traveled (mm) and average velocity (mm/s). **(C)**. The swimming behavior of costunolide (COS) and dehydrocostus lactone (DHE) obtained via zebrafish behavior trajectory analysis system are analyzed as total distance traveled (mm) and average velocity (mm/s). Data are expressed as the mean ± SEM (n = 8/group). Significance was defined as ^##^ *p* < 0.01, ^###^ *p* < 0.001,^*^ *p* < 0.05, ^**^ *p* < 0.01, ^***^ *p* < 0.001.

Based on the antidepressant effect exhibited by ASBX, we further screened its components. We tested the survival rate of zebrafish embryos by ASBX, PEE, DME, EAE, BUE and AQE to determine the toxicity of each extract. There was no significant difference in the survival rate between each extract and the Con group, which proved that each extract had no obvious toxicity at this concentration (Supplementary Figure S2). Under photoperiod (dark/light alternating) stimulation, neurotoxic phenotypes at higher doses of reserpine show decreased swimming behavior in the zebrafish larvae (Wang et al., 2019). Compared with the Mod group, PEE had the best activity among the extraction layers, in terms of total swimming distance and average swimming speed (Figure 3B). Therefore, the PEE layer was chosen for further identification and separation by HPLC-Q-TOF-MS/MS (Figure 3A), and the antidepressant activity of the 23 fractions was evaluated (Figure 3B). Among them, fractions 26 and 28 improved the swimming ability of zebrafish larvae depression model induced by reserpine. Fraction 26 corresponds to the substance flowing out in 25–27 min in the ion flow diagram, and stream 28 corresponds to the substance flowing out in 27–29 min. According to the mass spectrum information, we identified that the components contained in fraction 26 are costunolide and costic acid, and the components contained in fraction 28 are dehydrocostus lactone (See for mass spectrum information in Supplementary Table S1). The antidepressant activities of compounds 1 and 2 were again verified by light/dark preference test. Zebrafish larvae treated with COS or DHE showed improved swimming activity of zebrafish larvae induced by reserpine (Figure 3C). The results thereby showed that COS and DHE were potentially the antidepressant components of ASBX.

### Anti-Depressive Effect of COS on Zebrafish Behavior

**FIGURE 4:**
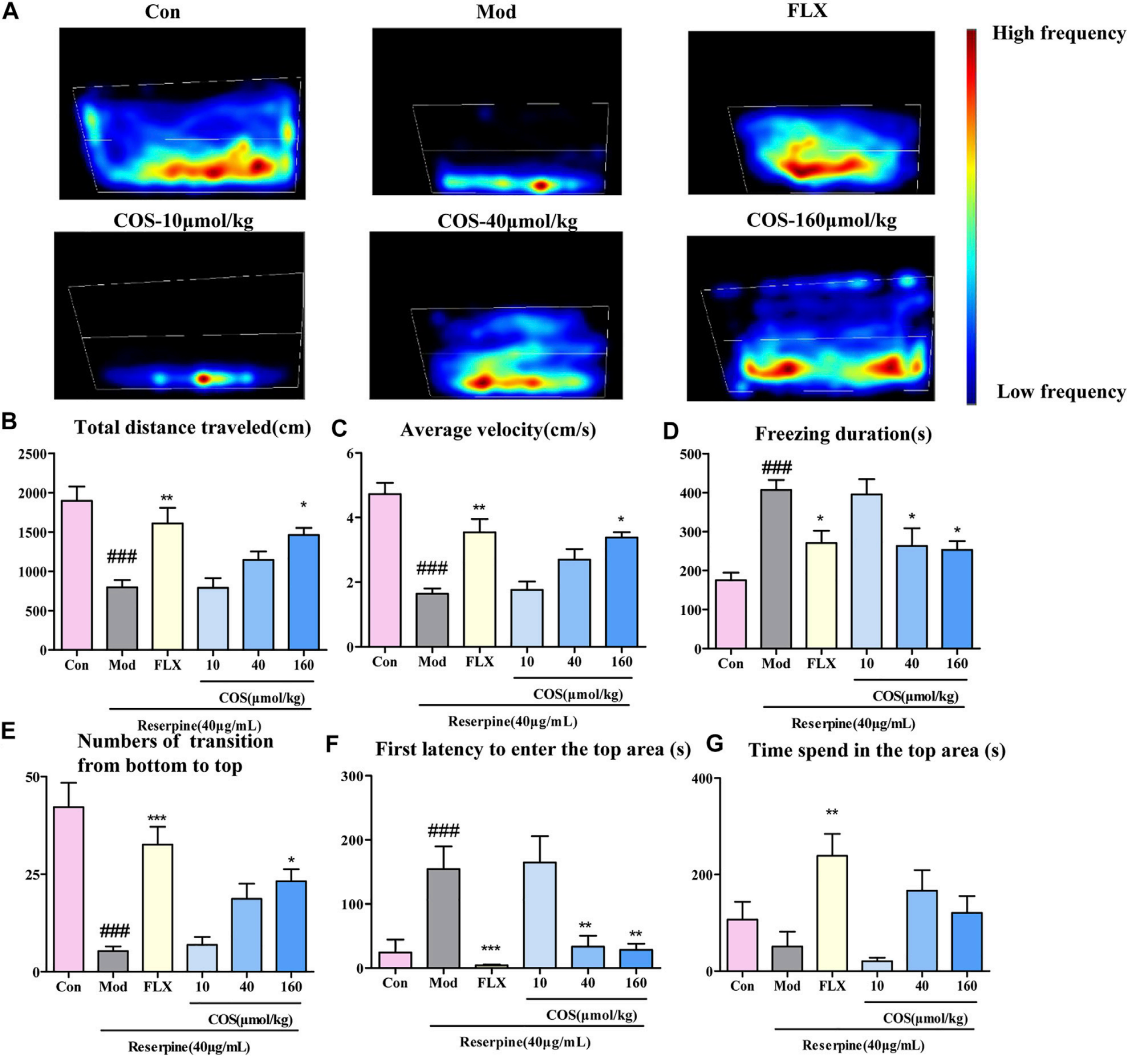
Antidepressant effect of costunolide (COS) was tested using a novel tank diving test. **(A)**. Heat maps of the behavior trajectory. The bar chart shows the swimming ability, freezing behavior and exploratory behavior of adult zebrafish. **(B)**. Total distance travelled (cm) **(C)**. Average velocity (cm/s) **(D)**. Freezing duration (s) **(E)**. Numbers of transition from bottom to top **(F)**. First latency to enter top area (s) **(G)**. Time spend in the top area Data are expressed as the mean ± SEM (n = 10/group). . Significance was defined as ^###^ *p* < 0.001 ^*^ *p* < 0.05, ^**^ *p* < 0.01, ^***^ *p* < 0.001.

To verify the antidepressant activity of COS, the NTDT was conducted, and the heat map shows that the depressive phenotype could be reversed by COS and FLX (Figure 4A). Compared with the Mod group, the COS-160 μmol/kg group showed improved swimming capacity (Figures 4B,C), freezing behavior (Figure 4D), and exploration behavior **(** Figures 4E,F). The COS-40 μmol/kg group showed improved depressive behavior with respect to freezing duration, first latency to enter the top (Figures 4D,F). There was no sigificant difference in time spend in the top area between the COS groups and Mod group (Figures 4G). The results showed that COS could improve the swimming behavior of zebrafish with reserpine induced depression.

### Protective Effect of COS on Reserpine-Induced Neuronal Injury

**FIGURE 5:**
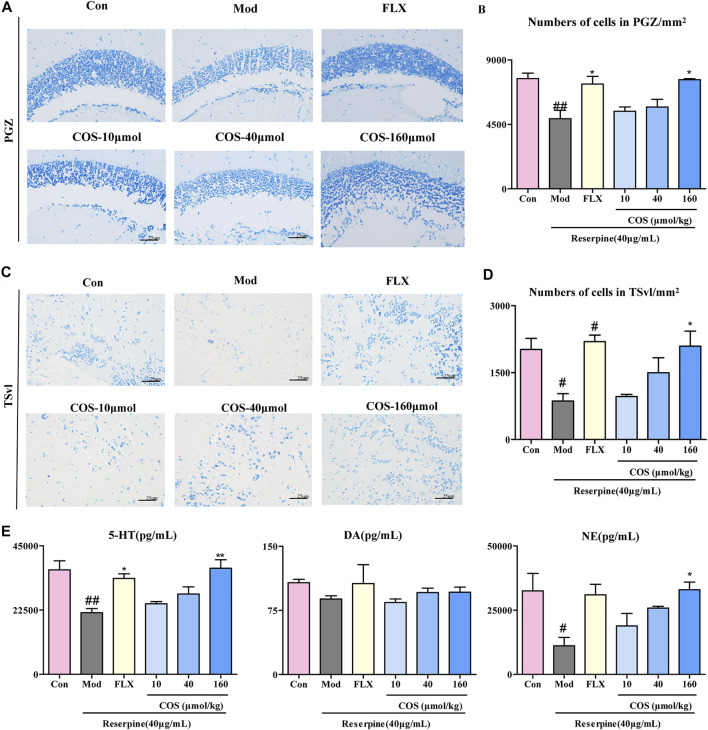
Protective effect of costunolide (COS) on reserpine-induced neuronal injury. **(A)**. Nissl stain of the zebrafish brain (400×) in periglomerular gray zone (PGZ) **(C)**. Nissl stain of the zebrafish brain (400×) in ventrolateral nucleus of the semicircular torus (TSvl). **(B and D)** are the quantitative statistics of neurons of the PGZ and TSyl, respectively. **(E)**. The content of 5-HT, DA, NE in zebrafish brain was measured via ELISA. Data are expressed as the mean ± SEM (n = 3/group). Significance was defined as ^#^ *p* < 0.05, ^##^ *p* < 0.01, ^*^ *p* < 0.05, ^**^ *p* < 0.01.

The anti-depression effect of COS was also verified at the histopathological and biochemical levels. The results of Nissl staining showed that the neuronal loss in PGA and TSvl in the Mod group was improved by COS (COS-160 μmol/kg) and FLX. (Figures 5A–D). Compared with the Mod group, in the COS-160 μmol/kg group, the levels of 5-HT and NE was increased. (Figure 5E). The results showed that cos had a protective effect on reserpine induced nerve injury, which may be related to the increase of 5-HT and NE.

### COS Decreased the Expression Level of SERT and NET

**FIGURE 6:**
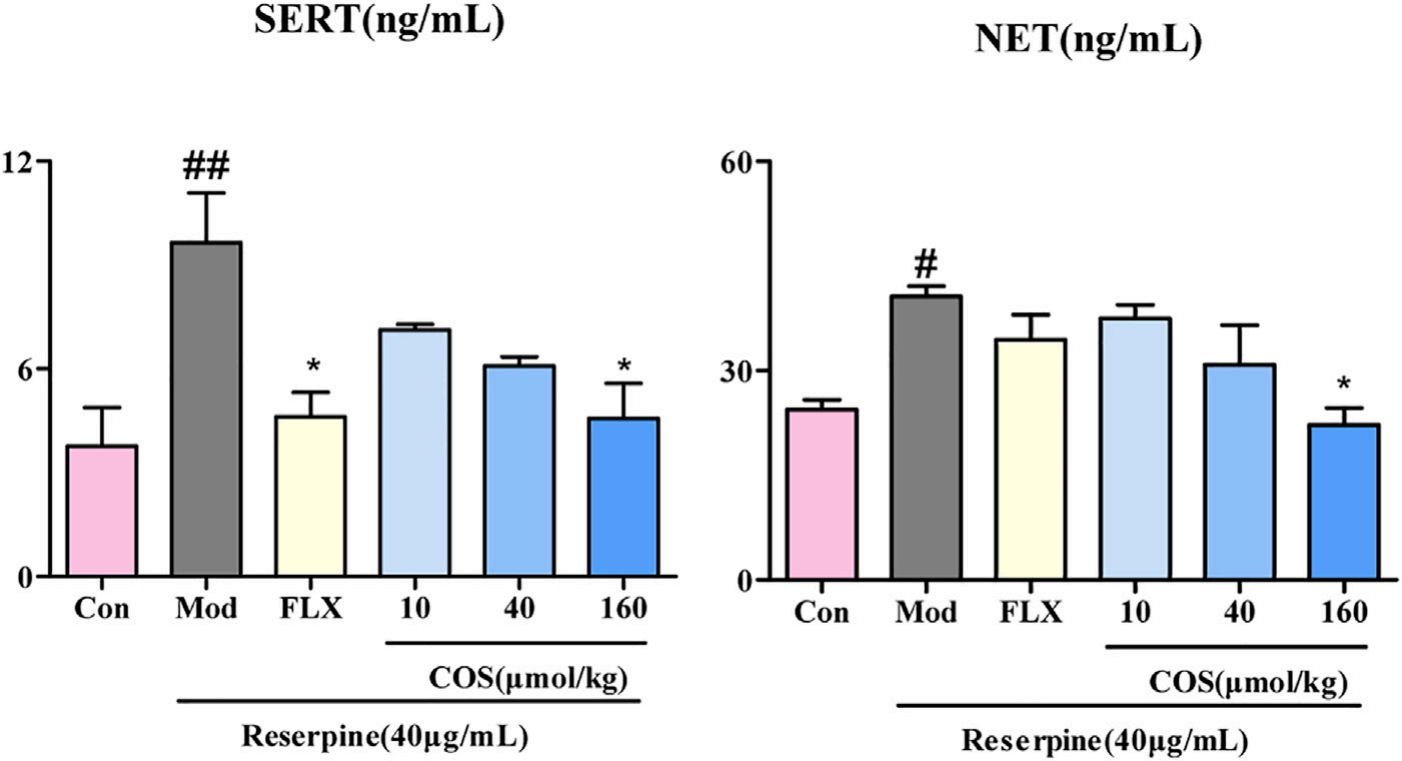
Effect of costunolide (COS) on the expression of serotonin transporter (SERT) and norepinephrine transporter (NET). The content of SERT and NET in zebrafish brain was measured via ELISA. Data are expressed as the mean ± SEM (n = 3/group). Significance was defined as ^#^ *p* < 0.05, ^##^ *p* < 0.01, ^*^ *p* < 0.05.

The upregulation of SERT and NET leads to a lack of 5-HT and NE in the synaptic cleft, which may be one of the causes of depression (Chen et al., 2012; Li et al., 2019). The COS-160 μmol/kg treatment showed inhibition of expression of SERT and NET compared with Mod group (Figure 6).

## Discussion

The active components of TCMs are usually complex and found in trace quantities (Liao et al., 2020). How to achieve rapid separation and enrichment of components in complex samples, and how to detect the activity on the basis of trace compounds, are problems that still need to be solved. In this study, a phenotype-based screening strategy, utilizing HPLC-Q-TOF-MS/MS technology combined with a zebrafish behavior trajectory analysis system, was established and used to screen the antidepressant components of ASBX. Firstly, we found the antidepressant effect of ASBX through the new tank diving experiment of adult fish. Secondly, a depression model of zebrafish larvae induced by reserpine was established, which showed a significant decline in swimming behavior (Figure 3B), which was consistent with the literature reports (Wang et al., 2019). Thirdy, the light/dark preference test was used to detect the activity of the components of ASBX separated by HPLC. The screened components showed the ability to affect the swimming behavior of zebrafish larvae at the micromolar range (Figures 3B,C), which indicates that the method can realize the activity detection of trace components. In addition, the zebrafish behavior trajectory analysis system can automatically image and digitally analyze the swimming behavior of a large scale of zebrafish larvae in microplates (i.e. 96 per plate and many plates consecutively), to evaluate the influence of multiple components on behavior function at the same time. Finally, the active components were identified as COS and DHE by HPLC-Q-TOF-MS/MS, Here, the antidepressant effect of DHE is reported for the first time. It is a sesquiterpene with a structure similar to COS. Volatile and fat-soluble components have been shown to be psychoactive (Brillatz et al., 2020). We speculate that the antidepressant activity of COS and DHE may be that these sesquiterpenoids are fat soluble components and easy to penetrate into the brain, so as to achieve the required concentration of antidepressants.

**FIGURE 7:**
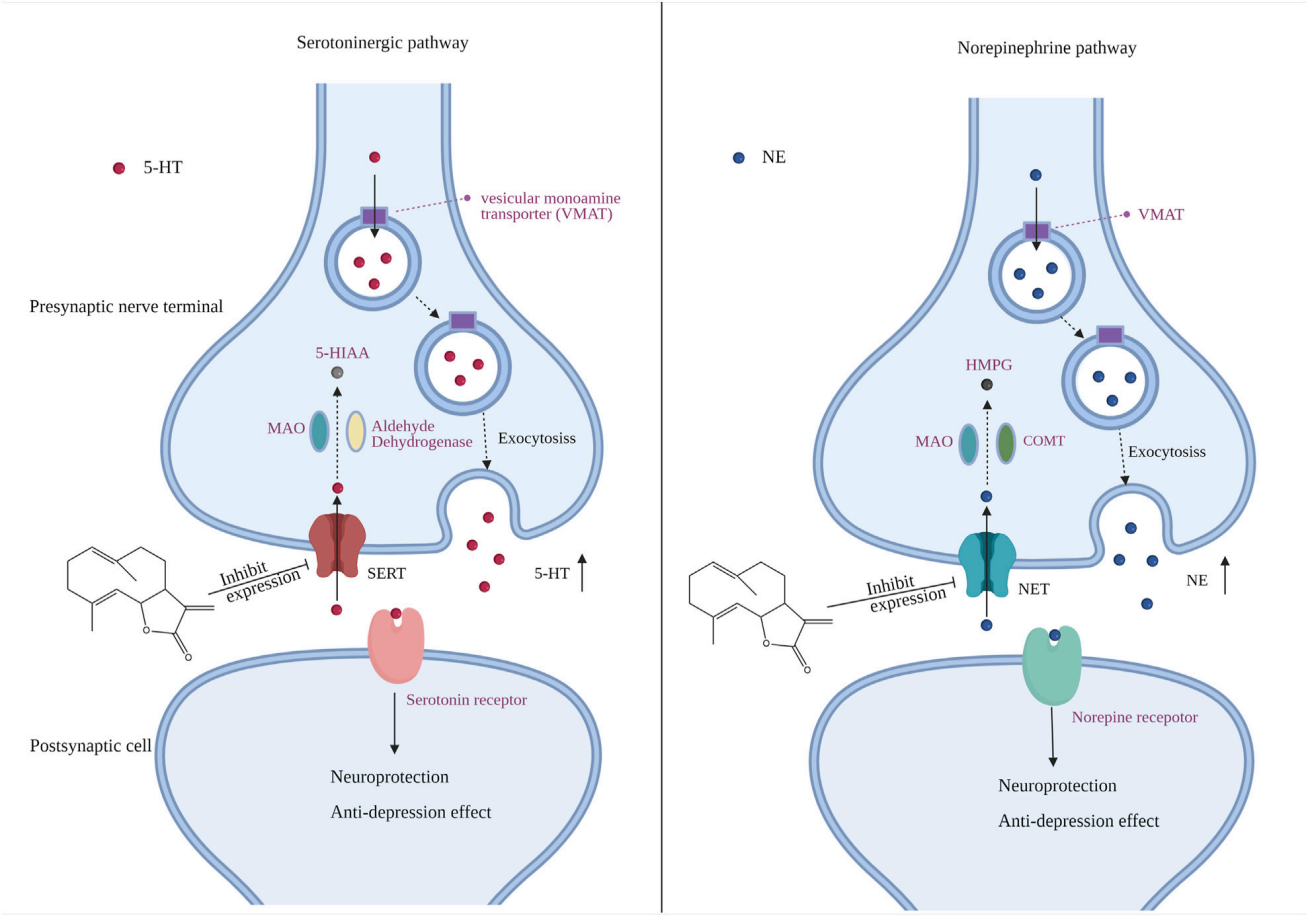
Potential mechanism of the antidepressant effect of costunolide (COS). Through inhibiting the expression of SERT and NET, COS inhibits the reuptake function of 5-HT and NE in the process of synaptic transmission, to increase the level of 5-HT and NE and activate related receptors, which is the potential mechanism of its antidepressant effect. The Figure 7 was created with BioRender.com.

Monoamine neurotransmitters such as serotonin (5-HT), DA and NE are widely distributed in the developing and adult central nervous system (CNS), affecting numerous physiological processes, including depression and anxiety (Wilhelm et al., 2008). Neurotransmitters such as 5-HT are synthesized by presynaptic neurons and stored in vesicles through vesicle transporters. They are transported to nerve endings through vesicles, exocytosis is released to synaptic space, activate postsynaptic receptors and stimulate postsynaptic neurons, resulting in changes in a series of signal pathways (Ochi et al., 2019). Reserpine is an inhibitor of vesicle monoamine transporter (Wilhelm et al., 2008), which inhibits the entry of neurotransmitters into vesicles. Our results indicated that COS improves the contents of 5-HT and NE in zebrafish brain tissue of reserpine induced depression model, and improved swimming behavior and nerve injury (Figure 5C, 6). These results also further confirmed the important role of 5-HT and NE system in the regulation of depression by the central nervous system (Figure 7). The inhibition of monoamine transporter can lead to its aggregation in synaptic space and prolong the activation time of receptor (Taciak et al., 2018). Therefore, transporters play a very important role in the transmission of neurotransmitters. SERT is a monoamine transporter,which can terminate 5-HT transmission through rapid presynaptic uptake (Orlando et al., 2020). The fuction of SERT may be related to several different mechanisms, such as the increase of expression of SERT (Li et al., 2019) and the change of SERT conformation (Plenge et al., 2020). NET is also a neurotransmitter transporter, which accelerates the inactivation of NE through the reabsorption of NE released from the synaptic space of central and peripheral nervous system (Fentress et al., 2013). Both SERT and NET belong to sodium/chloride dependent transporters. After binding to the substrate, sodium and chloride ions are transferred to the cytoplasm together with the substrate (Loland et al., 2003). The function of NET can be changed by many drugs. These drugs can directly bind to transporters, for example, NET after glycosylation will be expressed on the cell surface, so as to have functionality, or inhibit its function, such as changing the activity of cell membrane Na^+^/K^+^ adenosine triphosphatase, affecting the supply of energy during transport, acting as antagonists, or competing with substrates to change function (Mandela and Ordway, 2006). The knockout of SERT and NET reduced immobility in forced swimming test and tail suspension test (Kalueff et al., 2007; Solich et al., 2020). Chronic stress leads to the increase of content of SERT and NET, which may be the reason for the decrease of NE and SERT level in brain tissue (Pidathala et al., 2021). Therefore, regulating the expression of SERT and NET plays an important role in the occurrence of depression. Studies have shown that both corticotropin releasing factor (CRF) and CRF receptor agonists can increase the expression of NET (Huang et al., 2015). CRF is a neuropeptide that plays an important role in the regulation of hypothalamic pituitary adrenal axis (HPA) (Bijlsma et al., 2011). CRF receptor blockers can block the activation of noradrenergic neurons induced by stress, CRFR1 or CRFR2 antagonists can restore the expression of net to normal level and increase the level of NE (Huang et al., 2015), so as to further play a regulatory role in depression. The absence of SERT leads to obvious fear learning defect, and the blocker of CRF1 receptor can restore this phenotype to normal, which means the interaction between signal pathway of CRF and 5-HT in nervous system (Bijlsma et al., 2015). Previous studies have revealed that COS may form hydrogen bonds by occupying the allosteric sites of SERT, affecting the binding of 5-HT to the active pocket, so as to inhibit the reuptake of 5-HT (Li et al., 2021). In our study, COS can inhibit the high expression of SERT and NET induced by reserpine, which indicates that the inhibition of SERT and NET may be the potential mechanism of antidepressant effect of COS. In the next study, we may further study the antidepressant target of COS, which may be related to inhibition of CRF receptor.

Traditional screening methods can be divided into two categories, the method based on target and cell (Qian and Tcw, 2021), and the method based on animal (Paunovic et al., 2017). Affinity based biological separation methods, such as affinity chromatography (Krahn et al., 2020) and activity detection methods based on receptor (Gao et al., 2019) are used to characterize the active components and targets in traditional Chinese medicine. However, it is not suitable for screening active ingredients which target is unknown. The occurrence of depression is usually the result of multiple target regulation, which is usually characterized by behavioral phenotype. The efficacy of drugs in the nervous system cannot be explained at the level of cells and targets. Therefore, it is necessary to establish a phenotype based screening method.

Traditional mammalian based screening methods usually can not detect a large number of components at the same time and use a large amount of drugs (Geng et al., 2019). The antidepressant screening method of HPLC-Q-TOF-MS/MS technology combined with a zebrafish behavior trajectory screening system has significant advantages. Firstly, the method can detect the drug concentration at the micro molar level and can be used to detect trace samples. Secondly, this method can detect a large number of samples in 96 well plate at the same time, so it can realize the rapid screening of a large number of compounds. Finally, zebrafish larvae can sensitively show photokinetic response under strong light stimulation, and can respond to low concentration drug stimulation (Kokel et al., 2012). This method is simple and rapid for screening antidepressant components, but it still faces some challenges. The development of phenotype screening research relies more and more on the advancement of automatic video recording and digital behavior analysis systems. These systems have the purpose of analyzing and learning cognitive behavior through three-dimensional motion tracking. Digital behavior analysis can provide light, sound, heat and other signal stimulations (Henry and Wlodkowic, 2019). At the same time, it is necessary to enrich the behavior evaluation indexes of screening methods, such as establishing behavior fingerprints and behavior digital bar codes (Fuller et al., 2018), to further systematically and comprehensively analyze the pharmacodynamic characteristics of chemical components.

## Conclusion

In this study, the strategy of HPLC-Q-TOF-MS/MS technology combined with a zebrafish behavior trajectory screening system was successfully applied to screen and identify two antidepressant components, COS and DHE, from ASBX. The antidepressant effect of COS may be related to the inhibition of high expression of SERT and NET, the improvement of 5-HT and NE levels,and the repair of neuronal damage.

## Data Availability

The original contributions presented in the study are included in the article/Supplementary Material, further inquiries can be directed to the corresponding authors.

## Abbreviations

ASBX, Methanol extract of Anshen Buxin Six Pills; COS, Costunolide; DHE, dehydrocostus lactone; HPLC-Q-TOF-MS/MS, High-performance liquid chromatography coupled with quadrupole time-of-flight mass spectrometry; NTDT, Novel tank diving test; NE, Norepinephrine; NET, norepinephrine transporter; SERT, serotonin transporter; TCMs, traditional Chinese medicines; 5-HT, 5-hydroxytryptamine.
