# Supplementary material for: Phenotype-Based HPLC-Q-TOF-MS/MS Coupled With Zebrafish Behavior Trajectory Analysis System for the Identification of the Antidepressant Components in Methanol Extract of Anshen Buxin Six Pills
Source: Front Pharmacol. 2021 Nov 22;12:764388. doi: 10.3389/fphar.2021.764388 (PMC8645982; doi:10.3389/fphar.2021.764388)
Supplement: Supplementary file 1 [file DataSheet1.docx]

Supplementary Information for

**Phenotype-based HPLC-Q-TOF-MS/MS coupled with zebrafish behavior trajectory analysis system for the identification of the antidepressant components in methanol extract of Anshen Buxin Six Pills**

**Jiani Liu^1†^, Yue Shang ^2†^, Juanlan Xiao ^3^, Huirong Fan ^2^, Min Jiang ^1 *^, Saijun Fan ^2^ ^*^, Gang Bai ^1^**

^1^ State Key Laboratory of Medicinal Chemical Biology, College of Pharmacy and Tianjin Key Laboratory of Molecular Drug Research, Nankai University, Haihe Education Park, 38 Tongyan Road, Tianjin 300353, China

^2^ Institute of Radiation Medicine, Chinese Academy of Medical Sciences and Peking Union Medical College, 238 Baidi Road, Nankai District, Tianjin 300110, China

^3^ Graduate school, Tianjin University of Traditional Chinese Medicine, Tianjin 301617, China

**†**These authors have contributed equally to this work and share first authorship

*** Correspondence:**

Min Jiang,Tel and Fax:86-22-23506930, E-mail: minjiang@nankai.edu.cn

Saijun Fan,Tel and Fax: 86-22-85685301, E-mail: [fansaijun@irm-cams.ac.cn](mailto:fansaijun@irm-cams.ac.cn)

This file includes:

**Figure S1. Total ion chromatograms (TIC) of the petroleum ether extract (PEE) of ASBX in positive ESI mode**

**Figure S2.Effects of ASBX, PEE, DME, EAE, BUE, AQE on survival rate of zebrafish larvae.**

**Table S1. Identification of COS and DHE from petroleum ether extract (PEE) of Anshen Buxin Six Pills by HPLC-Q/TOF-MS in positive ESI mode.**

**Methods for Effects of ASBX, PEE, DME, EAE, BUE, AQE on toxicity of zebrafish larvae.**

**Figure S1. Total ion chromatograms (TIC) of the petroleum ether extract (PEE) of ASBX in positive ESI mode.**

**
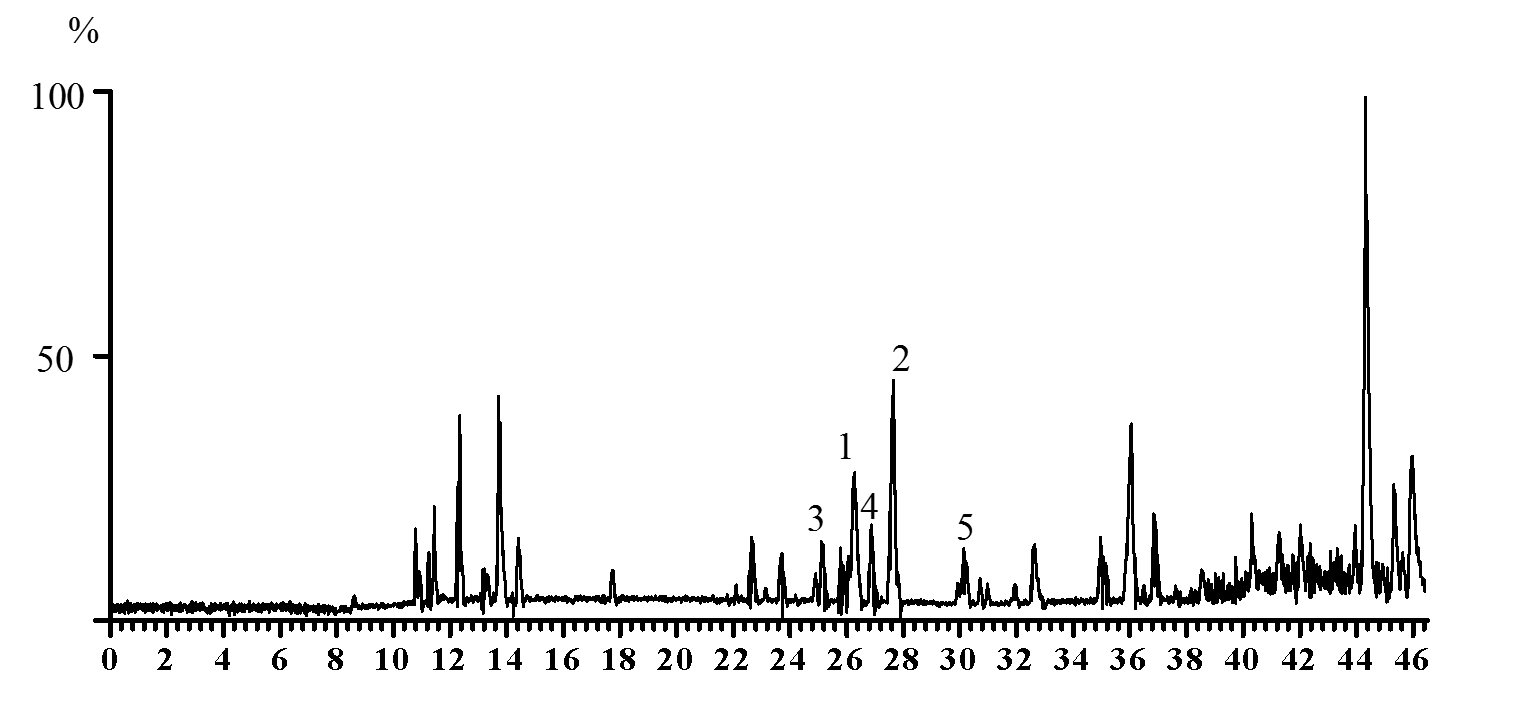
**

**Table S1.Identification of components from petroleum ether extract (PEE) of Anshen Buxin Six Pills by HPLC-Q/TOF-MS in positive ESI mode.**

| **N^o^** | **Retention**  **Time (min)** | **Observation. m/z [M+H]^+^** | **Calculated.m/z**  **[M+H]^+^** | **Error**  **(ppm)** | **Formula** | **MS/MS data m/z** | **Compound** |
| --- | --- | --- | --- | --- | --- | --- | --- |
| **1** | 26.28 | 233.1535 | 233.1536 | -0.42 | C_15_H_20_O_2_ | 215.1441;187.1487;145.1002;131.0861 | Costunolide ^(Peng et al., 2014)^ |
| **2** | 27.59 | 231.1376 | 231.1379 | -1.30 | C_15_H_18_O_2_ | 213.1276;185.1316;143.0853;129.0693 | Dehydrocostus lactone^(Peng et al., 2014)^ |
| **3** | 25.87 | 193.0856 | 193.0859 | -1.55 | C_11_H_12_O_3_ | 152.0474 | Myristicin ^(Pandey et al., 2016)^ |
| **4** | 26.73 | 235.1692 | 235.1688 | 1.70 | C_10_H_12_O_2_ | 161.0119 | Costic acid ^(He et al., 2018)^ |
| **5** | 30.24 | 327.1579 | 327.1591 | -3.67 | C_20_H_22_O_4_ | 203.1029;177.0994 | Dehydrodiisoeugenol ^(Zhang et al., 2021)^ |

**Methods for Effects of ASBX, PEE, DME, EAE, BUE, AQE on toxicity of zebrafish larvae.**

Freeze-dried extraction layers different extraction layers (PEE, DME, EAE, BUE, AQE ) and lyophilized powder of ASBX were dissolved with DMSO and diluted with Holt buffer to the content of DMSO was 0.1%. The experimental groups are described as follows: Con group (0.1% DMSO Holt Buffer ), different extraction layers (PEE, DME, EAE, BUE, AQE, 80 μg/mL), ASBX methanol extract (80 μg/mL) in 12-well plates. 30 eggs were placed in each well, and 3 repeated controls were set in each group. The survival rate of fish embryos were counted at 24, 48, 72 hours after administration.

**Figure S2.Effects of ASBX, PEE, DME, EAE, BUE, AQE on survival rate of zebrafish larvae.** GraphPad Prism Software (version 6.0) was used for the statistical analysis of results, and two-way analysis of variance was applied for data analysis Data are expressed as the mean ± SEM (n = 3/group).


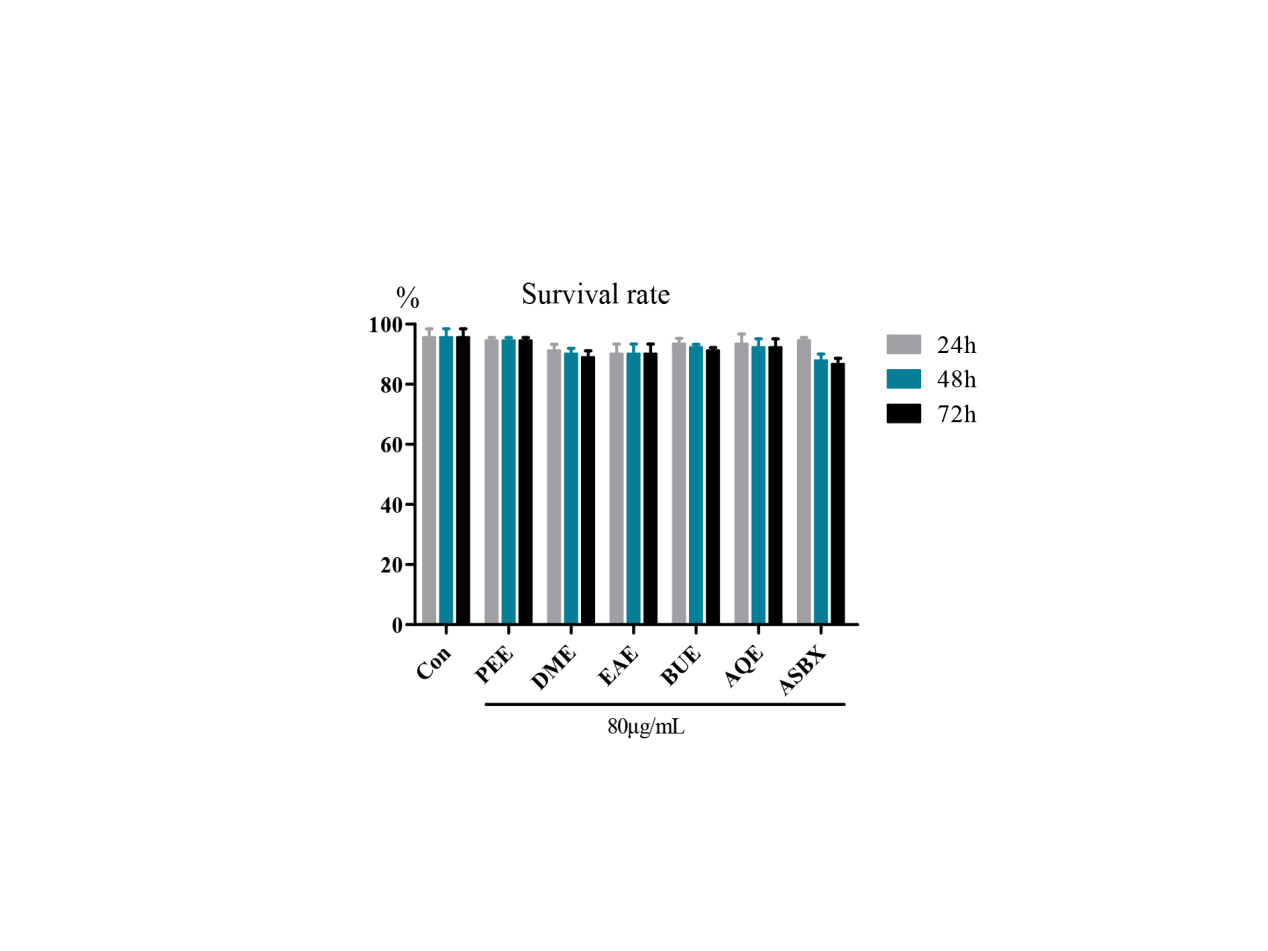


He, Y., Cheng, P., Wang, W., Yan, S., Tang, Q., Liu, D., et al. (2018). Rapid Investigation and Screening of Bioactive Components in Simo Decoction via LC-Q-TOF-MS and UF-HPLC-MD Methods. *Molecules* 23(7). doi: 10.3390/molecules23071792.

Pandey, R., Mahar, R., Hasanain, M., Shukla, S.K., Sarkar, J., Rameshkumar, K.B., et al. (2016). Rapid screening and quantitative determination of bioactive compounds from fruit extracts of Myristica species and their in vitro antiproliferative activity. *Food Chem* 211**,** 483-493. doi: 10.1016/j.foodchem.2016.05.065.

Peng, Z., Wang, Y., Gu, X., Guo, X., and Yan, C. (2014). Study on the pharmacokinetics and metabolism of costunolide and dehydrocostus lactone in rats by HPLC-UV and UPLC-Q-TOF/MS. *Biomed Chromatogr* 28(10)**,** 1325-1334. doi: 10.1002/bmc.3167.

Srivastava, D., and Cohen, D.E. (2009). Identification of the Constituents of Balsam of Peru in Tomatoes. *Dermatitis* 20(2)**,** 99-105. doi: 10.2310/6620.2008.08008.

Zhang, J., Si, H., Sun, J., Lv, K., Yan, B., Li, B., et al. (2021). Determination of myrislignan levels in BALB/c mouse plasma by LC-MS/MS and a comparison of its pharmacokinetics after oral and intraperitoneal administration. *BMC Vet Res* 17(1)**,** 275. doi: 10.1186/s12917-021-02990-y.

**References**
